# CMR assessment of longitudinal left ventricular function following transcatheter aortic valve implantation (TAVI) for severe aortic stenosis

**DOI:** 10.1186/1532-429X-17-S1-P180

**Published:** 2015-02-03

**Authors:** Laura E Dobson, Tarique A Musa, Timothy A Fairbairn, Akhlaque Uddin, Daniel J Blackman, David P Ripley, Peter P Swoboda, Adam K McDiarmid, Bara Erhayiem, Pankaj Garg, Sven Plein, John P Greenwood

**Affiliations:** Multidisciplinary Cardiovascular Research Centre & Leeds Institute for Cardiovascular and Metabolic Medicine, University of Leeds, Leeds, UK; Yorkshire Heart Centre, Leeds Teaching Hospitals Trust, Leeds, UK

## Background

Global longitudinal strain and mitral annular plane systolic excursion (MAPSE) have been validated as an echocardiographic measure of longitudinal function in patients with aortic stenosis (AS). Cardiac magnetic resonance imaging (CMR) offers a simple and easy measure of MAPSE and been validated in various patient groups. Longitudinal function in aortic stenosis is usually reduced before a reduction in ejection fraction is seen. We sought to investigate the impact of TAVI upon longitudinal function and global ejection fraction in patients treated for symptomatic severe AS.

## Methods

**Figure 1:**
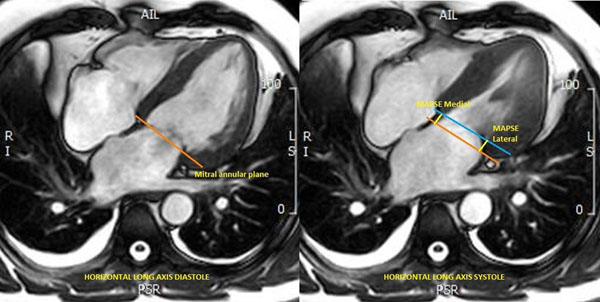
Method for calculation of mitral annular systolic plane excursion

We prospectively enrolled 52 patients with symptomatic severe aortic stenosis undergoing TAVI over a 5 year period from March 2009 to March 2014. Patients with contraindications to CMR were excluded and all patients provided informed written consent. All patients underwent an identical 1.5T CMR protocol (Intera, Phillips Healthcare, Best, The Netherlands) at baseline and at a median of 6 months following TAVR. Multi-slice, multi-phase cine imaging was performed using a standard steady-state free procession pulse sequence in the short and long axis (8 mm thickness, 0 mm gap, 30 phases, typical field of view (FOV) 340 mm) to cover the entire left and right ventricle. Longitudinal atrioventricular motion was measured at the septal and lateral mitral annulus in the 4 chamber cine view (Figure 1) at end systole and end diastole. Offline analysis (CVI42, Circle Cardiovascular Imaging, Calgary, Alberta, Canada) included measuring the perpendicular distance between these two points.

## Results

52 patients (mean age 80.3 ± 6.8 years, 30 (58%) male, AVA 0.6 ± 0.2 cm^2^, Logistic Euroscore 20.1 ± 13.9%) were studied. There was no significant change in the overall LV ejection fraction 6 months following TAVI compared to baseline. All three measures of longitudinal function (MAPSE^Lateral^, MAPSE^Medial^ and MAPSE^Average^) were significantly improved 6 months following TAVI (Table 1). There was a moderate correlation between all three MAPSE values and LV ejection fraction (MAPSE^lateral^ r=0.38, p=0.005, MAPSE^medial^ r=0.42, p=0.002 and MAPSE^Average^ r=0.44, p=0.001). Presence or absence of late gadolinium enhancement did not impact on the baseline MAPSE^average^ (9.7mm Vs 9.1mm, p=0.44) or have impact on the change in MAPSE^average^ following TAVI (0.7mm Vs 1.3mm, p=0.48).

**Table 1:** Mean LV ejection fraction and MAPSE values before and 6 months after TAVI. Values expressed as mean ± SD.

	Pre-TAVI	Post-TAVI	P Value
LV ejection fraction, %	54.9 ± 13.2	56.8 ± 11.9	0.08
MAPSELateral, mm	10.6 ± 2.9	11.7 ± 3.1	0.005
MAPSEMedial, mm	8.5 ± 2.4	9.2 ± 2.4	0.04
MAPSEAverage((medial + lateral) / 2), mm	9.6 ± 2.4	10.5 ±2.6	0.005

## Conclusions

Longitudinal left ventricular function, as assessed using MAPSE, improved following TAVI for severe aortic stenosis. MAPSE may be a more sensitive marker of left ventricular recovery following TAVI than global LV ejection fraction.

## Funding

This study was part funded by the British Heart Foundation (PG/11/126/29321).

